# Antibiotic resistance among Aerobic Gram-Negative Bacilli isolated from patients with oral inflammatory dysbiotic conditions—a retrospective study

**DOI:** 10.3389/fdmed.2024.1293202

**Published:** 2024-03-04

**Authors:** A. Basic, S. Blomqvist, G. Charalampakis, G. Dahlén

**Affiliations:** Department of Oral Microbiology and Immunology, Institute of Odontology, Sahlgrenska Academy, University of Gothenburg, Gothenburg, Sweden

**Keywords:** oral mucosa, periodontitis, peri-implantitis, Gram-negative aerobic bacteria, antibiotic resistance, Extended Spectrum Beta-Lactamase (ESBL)

## Abstract

**Introduction:**

Aerobic gram-negative bacilli (AGNB) are not part of the resident oral microflora but are occasionally found in high abundance under inflammatory dysbiotic conditions at various oral niches. The aim of the present study was to investigate the identity and antibiotic susceptibility of AGNB isolated from patients in Sweden with mucosal lesions, periodontitis, and peri-implantitis, with special attention to antibiotic resistance and on the presence of phenotypic Extended Spectrum Beta-Lactamase (ESBL) isolates.

**Materials and methods:**

Microbiolgical samples were harvested from 211 patients in total, experiencing mucosal lesions (*N* = 113), periodontitis (*N* = 62), or peri-implantitis (*N* = 36). The growth of AGNBs was semiquantified by selective and non-selective culture and the strains were isolated, identified, and tested for antibiotic susceptibility. A total of 251 AGNB strains, occurring in moderate to heavy growth (>100 CFU/ml sample), indicating a dysbiotic microbiota, were identified. The disc diffusion method was used for screening of the antibiotic susceptibility of the isolates. Phenotypic identification of ESBL isolates was based on resistance to ceftazidime and/or cefotaxime.

**Results:**

The most commonly detected AGNB isolates in oral inflammatory dysbiotic conditions were fermentative species belonging to *Enterobacteriaceae* e.g. *Citrobacter* spp.*, Enterobacter* spp.*, Escherichia coli, Klebsiella* spp*,* and the non-fermentative environmental *Burkholderia cepacia, Pseudomonas* spp., and *Stenotrophomonas maltophilia*. No clear trends were seen in frequency of the various species in samples from mucosal lesions, severe periodontitis, and peri-implantitis cases. The 138 *Enterobacteriaceae* isolates and 113 environmental AGNB isolated showed a high antibiotic resistance in general against antibiotics commonly used in dentistry (Amoxicillin, Amoxicillin + Clavulanic acid, Ampicillin, Clindamycin, Doxycycline, Erythromycin, Oxacillin, PenicillinV, and Tetracycline). The majority of these isolates were susceptible to ciprofloxacin. Ten isolates (4.1%) were phenotypically classified as ESBL positive. The ESBL isolates were predominantly found among isolates of *S. maltophilia*, while only one ESBL positive isolate was found among *Enterobacteriaceae*.

**Conclusions:**

Phenotypically identified ESBL isolates can occasionally be present among oral AGNB strains isolated in abundance from the dysbiotic microbiota occurring in cases with oral mucosal lesions, severe periodontitis, or peri-implantitis.

## Introduction

Aerobic Gram-negative bacteria, bacilli/rods (AGNB), are frequently detected in urinary tract infections (UTI), respiratory tract infections, and various opportunistic infections in hospitalized and immune-compromised patients globally (1–3). They are generally multidrug resistant and the increasing spread of ESBL (Extended Spectrum Beta-Lactamase) with remarkable resistance against the third-generation cephalosporins, is an emerging problem in bacterial infections worldwide (4).

AGNBs are normally considered as non-oral, although they may sporadically occur in low numbers in healthy patients and as such, be present in the transient oral microbiota (5–8). In immune-compromised and multi-diseased patients however, AGNBs are quite frequently the opportunists of oral infections. Although fungal infections are the most common in mucosal lesions (9), bacterial dysbiosis may also develop in patients with systemic immune-compromised conditions favoring an ecological change and an imbalance (dysbiosis) leading to adaptation and overgrowth of opportunistic bacteria such as *Staphylococcus aureus*, *Enterococcus faecalis* and various AGNBs (10, 11). In a recent overview of the presence of non-oral bacteria in the oral cavity, the prevalence of AGNBs, although sparsely studied, differs substantially between various populations (8). In Sweden, a retrospective study including patients with oral infections and mucosal complaints reported that moderate to heavy growth of yeasts (Candida species) was detected in 52.2% of the cases, while bacteria such as *S. aureus*, enterococci and AGNBs were found in 12.5%, 11.1% and 35% respectively (12).

Local compromised conditions such as dry mouth, mucosal atrophia and/or hyperkeratosis, with concomitant mucosal lesions and symptoms such as mucositis, burning sensation, and pain constitute ecological conditions for the establishment and growth of opportunistic bacteria (12). Correspondingly, opportunistic bacteria including AGNBs have in a similar manner been reported in local inflammatory conditions such as periodontitis and peri-implantitis (13, 14). The potential etiological role of opportunistic microorganisms in these local inflammatory dysbiotic conditions remains uncertain.

The AGNBs found in the dysbiotic oral conditions are heterogenous and include various species such as the species of the carbohydrate-fermenting family *Enterobacteriaceae* (commonly referred to as enteric rods) and the carbohydrate non-fermenting family *Pseudomonadaceae*. The bacteria are present in an abundance, a condition that indicates an overgrowth (dysbiosis) at the sampling site. The evidence of the presence of oral isolates of fermenting and non-fermenting AGNBs, their identification on species level, and their antibiotic resistance pattern is scarce (12, 14, 15). Antibiotic resistance is of specific concern in dentistry due to the common use of beta-lactam antibiotics in the treatment of periodontitis and peri-implantitis. Increased overall use of antibiotics, including the use in dentistry, has been associated with the emergence of beta-lactamases mediated bacterial resistance, which subsequently has led to the development of resistance against clavulanic acid, an inhibitor of beta-lactamases and ESBL-producing bacteria. ESBL, which are resistant against the third generation of cephalosporins, have been reported worldwide in many different genera of *Enterobacteriaceae* (*Escherichia coli* and *Klebsiella* spp. in particular) and *Pseudomonas* spp. (16, 17). While ESBL are frequently detected among various infections, such as UTI and respiratory tract infections (18), the presence of ESBL isolates of AGNBs from oral sites is to our knowledge only considered in three previous reports, one examining supragingival plaque, another on subgingival plaque, and a third sampling the dorsum of the tongue (15, 19, 20).

The aim of this study was to identify AGNBs and determine their antibiotic susceptibility profile among isolates from patients with mucosal complaints, periodontitis, and peri-implantitis, with special attention to the presence of ESBL isolates.

## Material and methods

### Patients and samples

All incoming mucosal samples to the Oral Microbiological Diagnostic Laboratory at the Department of Oral Microbiology and Immunology, Institute of Odontology, University of Gothenburg, Sweden for microbiological diagnostics of various oral lesions during a 5-year period (2007–2011) showing at least moderate growth (>100 CFU/ml sample) of AGNB were investigated (21). To exclude the potential transient presence of AGNB, samples with sparse growth of AGNB were excluded. The mucosal samples were harvested from 1,231 patients with general oral symptoms (burning sensation and mucositis) by scraping the tongue or, in the case of localized lesions, scraping the lesions. The reasons for taking the samples were either complaints of the patient or clinical diagnosis of a general stomatitis, an abnormal appearance or localized white or red lesions of the mucosa. The scrapings were transferred to transport medium VMGA III (22) and sent to the laboratory. The samples were processed in the laboratory as described in detail previously (12). Briefly the samples were streaked on selective and non-selective agar plates and were incubated aerobically with 10% CO_2_ for 1–2 days and anaerobically in anaerobic jars for 5–7 days. The plates were examined for typical colony morphology and were semi-quantified according to a scale published previously (12, 21). Aerobic Gram-negative rods present in moderate growth (>100 CFU) were pure cultured and stored at −80°C until identification.

Isolates from periodontitis cases were collected from incoming samples (*N* = 110) to the laboratory during 2008–2013 for diagnostic evaluation by culture. The subgingival samples were taken with paper points and transferred to transport medium VMGAIII (22).

The isolates from peri-implantitis cases were obtained from 100 patients (179 implants) who were treated with surgeries with/without adjunctive systemic and/or local antimicrobial therapy and were followed for 12 months (23). Samples were taken with paper points from peri-implantitis sites at baseline before treatment, and after 3, 6, and 12 months as previously described (23).

Samples from periodontitis and peri-implantitis cases were analyzed by culture for evaluation of the presence of black-pigmented Gram-negative anaerobic rods (*Porphyromonas gingivalis* and *Prevotella intermedia*) and *Aggregatibacter actinomycetemcomitans* and concomitantly showing an overgrowth of aerobic Gram-negative rods as previously described in detail (24). Briefly, the samples were diluted (1:100, and 1:10 000) and spread onto Brucella blood agar plates and TSBV (Trypticase soy with bacitracin and vancomycin) agar plates, incubated anaerobically as described by Charalampakis et al. (25). Isolates were stored at −80°C until further identification.

### Bacterial identification

The samples were incubated on MacConkey agar plates for 2–3 days specifically for differentiation of AGNBs into lactose and non-lactose fermenting species and this was additionally confirmed by the lactose test (26). Tests for oxidase, catalase, and the ability to reduce nitrate were performed according to Lennette et al. (26). Identification was further performed using the commercially available systems; API RapiD20E (Biomerieux, St Louis, MO, US) was used to identify *Enterobacteriaceae* in 4 h, while API 20 NE (Biomerieux) was used for lactose negative isolates. Bacterial suspensions were prepared in 0.85% NaCl for inoculation into the 8 conventional substrates and in AUX medium (Biomerieux) for inoculation into the 12 assimilation cupules. The tests were incubated for 24 h at 30°C.

### Antibiotic resistance

Routine screening for antibiotic susceptibility was performed using blood agar plates and the disc diffusion method (Oxoid, Basingstoke, UK) against 9 antibiotics commonly used in dentistry in Northern European Countries: Amoxicillin (AML), Amoxicillin + Clavulanic acid (AMC), Ampicillin (AMP), Clindamycin (DA), Doxycycline (DO), Erythromycin (E), Oxacillin (Ox), PenicillinV (P), and Tetracycline (TE) (27). Metronidazole, although frequently used in dentistry, was not included due to its inefficiency on aerobic or facultative bacteria. After incubation, the diameter of the inhibition zone of each strain was measured and the strains were graded as sensitive (S), or resistant (R) (28). In addition, antibiotic susceptibility was investigated against first generation cephalosporins (Cefadroxil (CFR), Cephalexin (CL)), second generation cephalosporin [Cefuroxime (XM)], third generation cephalosporins (Cefotaxime (CTX), Ceftibuten (CFT), Ceftazidime (CAZ)), fluoroquinolones (Ciprofloxacin (CIP), Norfloxacin (NOR)), and aminoglycoside [Gentamycin (CN)] using disc diffusion methods (Biomeriux).

Screening test for ESBL production was performed according to guidelines from the EUCAST using discs with CTX (30 µg) and CAZ (30 µg) placed on the media with the test inoculum and incubated for 24 h (29). Bacterial isolates showing CTX <19 mm and CAZ <19 mm zones, or resistance to CTX and/or CAZ using E-test, were considered to be potential ESBL producers. For confirmation of ESBL, E-test ESBL (AB BIODISK, Solna, Sweden) was performed in accordance with the instructions of the manufacturer. The test consisted of E-test with CTX/CTX + clavulanic acid and CAZ/CAZ + clavulanic acid. The isolates showing non-determinable results were further tested with cefepime/cefepime + clavulanic acid. Also, *Enterobacter* spp. and *Citrobacter* spp. were tested with cefepime/cefepime + clavulanic acid according to the guidelines from EUCAST (29).

## Results

### Patients and samples

Altogether 251 strains of Gram-negative bacilli were isolated from the analyzed samples (9.2% of incoming mucosal samples and 56.3% of the selected periodontitis samples were positive for AGNB). Ten percent of the peri-implantitis cases showed AGNB at baseline, as previously reported (23). The samples were harvested from individuals 6 to 96 years of age, mean age slightly above 60 years (Table 1). Females were overrepresented among patients with mucosal lesions and periodontitis, while there was a majority of men in the peri-implantitis group. The general health status of the patients was mainly unknown except for the mucosal lesion group, where the dentist had reported immune-compromised conditions such as cancers in 10 cases, and occasionally (2 cases) treatment with radiation therapy. In the periodontitis group, no data were available on the patient´s general health, while no immune-compromised patients were included in the peri-implantitis group, as previously reported (23).

**Table 1:** Characteristics of the individuals sampled and the number of isolates of AGNB.

Variable	Mucosa	Periodontitis	Peri-implantitis	Total
Number of patients (%)	113 (53.6)	62 (29.4)	36 (17.1)	211
Females (%)^a^	69 (62.7)	41 (68.3)	15 (42.9)	125 (61.0)
Age mean ± SD^a^	62.2 ± 21.2	61.1 ± 16.9	64.9 ± 17.0	62.3 ± 19.3
Median	66.0	65.5	68.0	66.0
Range	6–96	30–84	23–88	6–96
Number of immune-compromised/diseased (%)^b^	Cancer 10 (8.8) Radiation therapy 2 (1.8)	NA	0	
Number of isolated AGNB strains (%)	129 (51.4)	66 (26.3)	56 (22.3)	251

^a^ Data missing for 3 (mucosa), 2 (periodontitis) and 1 (peri-implantitis) individuals respectively.

^b^ No information reported for 79 mucosa, and all periodontitis individuals.

In total 129 strains were isolated from 113 samples of patients with various oral symptoms (Table 1). In 1 case 3 different strains were isolated from the same sample, and in another 14 cases 2 different strains were isolated from the same sample. Further, 66 strains from 62 samples from periodontal lesions (4 samples contained 2 different strains) were identified. In the peri-implantitis group, which was followed for 12 months and sampled at four occasions, strains at a later occasion were included only if they were not detected at baseline. In nine samples two strains were isolated, and in four cases three different strains were identified. Additionally, in three patients two different strains we identified at different occasions. Thus, 56 strains from 36 patients were isolated in the peri-implantitis group (Table 1).

The majority of the mucosal samples were from the dorsum of the tongue. Other common sample sites were open bone lesions, lips, and the palate. The harvesting location of 26.4% of the mucosal samples was not specified by the referring dentist (Table 2).

**Table 2:** Characteristics of the type of lesion and sampling location for the 251 isolates.

Location of sampling	Mucosa *N* = 129 (%)	Periodontitis *N* = 66 (%)	Peri-implantitis *N* = 56 (%)	Total *N* = 251 (%)
Dorsum of the tongue	43 (33.3)	–	–	43 (17.1)
Deep lesion, pus, bone	12 (9.3)	–	–	12 (4.8)
Lips, angle of the mouth	10 (7.8)	–	–	10 (4.0)
Palate	10 (7.8)	–	–	10 (4.0)
Buccal mucosa	9 (7.0)	–	–	9 (3.6)
Pharynx, tonsils	4 (3.1)	–	–	4 (1.6)
Vestibulum, saliva, plaque, skin	4 (3.1)			4 (1.6)
Gingiva	2 (1.6)			2 (0.8)
Periodontal pocket (%)	–	66 (100)	–	66 (26.3)
Peri-implant pocket (%)	1 (0.8)	–	56 (100)	57 (22.7)
Not defined	34 (26.4)			34 (13.5)

### Bacterial identification

**Figure 1:**
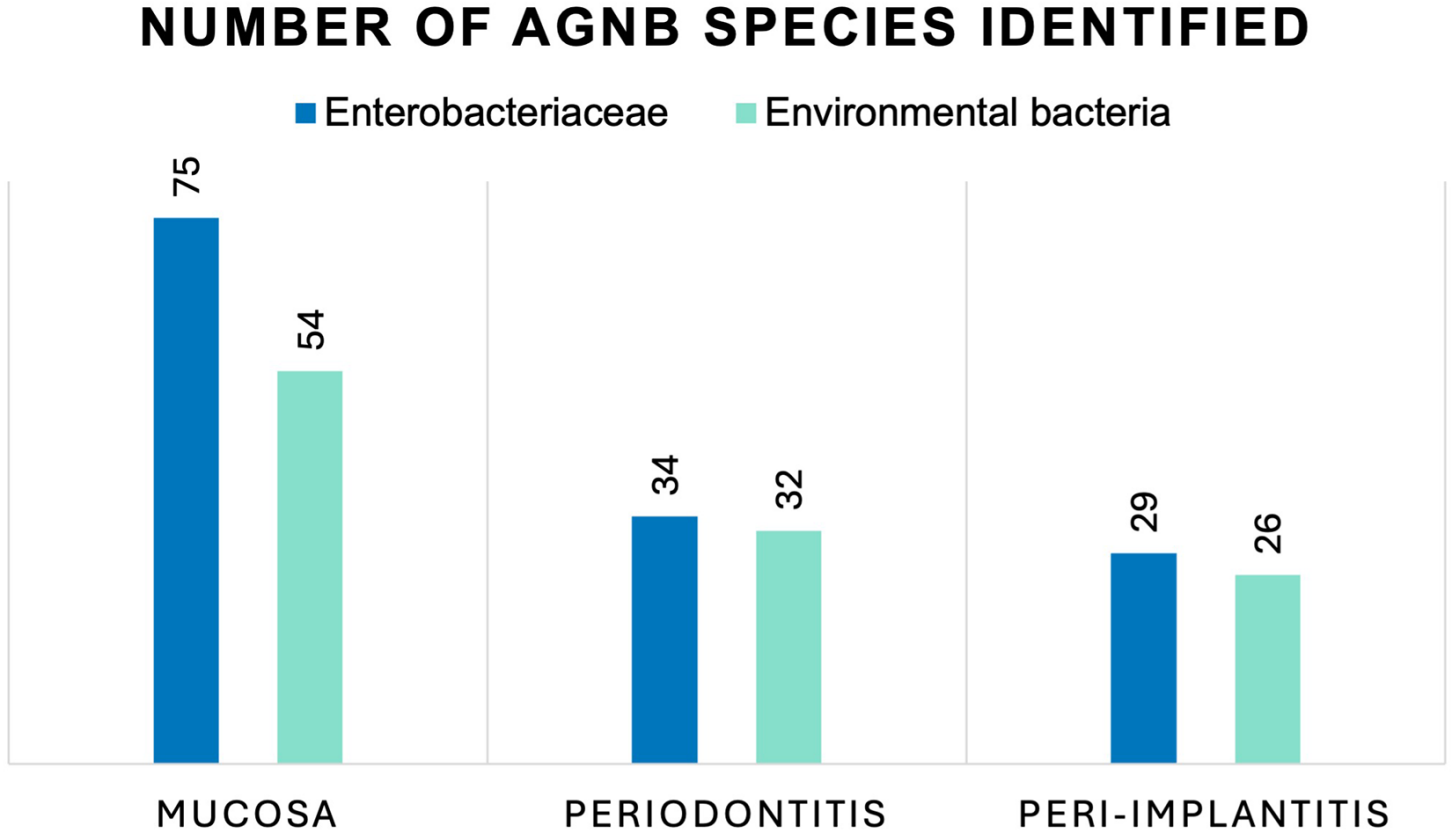
Number of AGNB species isolated from oral mucosal lesions, deep periodontal pockets, and peri-implant lesions identified as *Enterobacteriaceae* and environmental bacteria. No statistically significant differences were seen between the number of *Enterobacteriaceae* and environmental bacteria in the three groups, using Chi square test for independence (*p* = 0.62).

The phenotypic identification specified 251 isolates into species level (Table 3). Fifteen isolates were classified to species level only with uncertainty and were thus, classified only to genus level. Using API, it was not possible to identify one isolate harvested from a peri-implantitis lesion. The outcome showed a widespread classification, and no apparent trend or pattern was found for the samples from the three sampled groups (Figure 1). The most common genera found were *Klebsiella* (65 isolates), *Pseudomonas* (36 isolates), and *Stenotrophomonas* (21 isolates) in all three patient categories. *Enterobacter* spp. were also frequently found (24 isolates), mainly among the mucosal samples. The most common species isolated were *Klebsiella pneumoniae* (15.1%), *Klebsiella oxytoca* (10.8%), *Stenotrophomonas maltophilia* (8.4%), *Enterobacter cloacae (*8.0%), *Pseudomonas luteola* (6.8%), *E. coli* (6.4%), *Burkholderia cepacia* (6.0%), *and Pseudomonas aeruginosa* (4.8%). Some of the bacteria found have not been reported previously from the oral cavity, such as *Cronobacter* spp., *Cedecea davisae*, *Rhizobium radiobacter*, *Sphingomonas paucimobilis*, *Sphingobacterium* spp., *Delftia acidovorans* and *Cryseobacterium indologenes*.

**Table 3:** Non-oral AGNB species isolated from oral mucosal lesions, deep periodontal pockets, and peri-implant lesions.

AGNB species	Mucosa *N* = 129 (%)	Periodontitis *N* = 66 (%)	Peri-implantitis *N* = 56 (%)	Total *N* = 251 (%)
*Enterobacteriaceae* (enteric bacteria)				
*Cedecea davisae*	1 (0.8)	–	–	1 (0.4)
*Citrobacter braakii*	–	2 (3.0)	–	2 (0.8)
*Citrobacter freundii*	3 (2.3)	2 (3.0)	5 (8.9)	10 (4.0)
*Citrobacter koseri/amalonaticus*	2 (1.6)	–	–	2 (0.8)
*Cronobacter* spp.	–	1 (1.5)	–	1 (0.4)
*Enterobacter aerogenes* ^a^	1 (0.8)	–	1 (1.8)	2 (0.8)
*Enterobacter cloacae*	13 (10.1)	4 (6.1)	3 (5.4)	20 (8.0)
*Enterobacter* spp.	2 (1.6)	–	–	2 (0.8)
*Escherichia coli*	9 (7.0)	4 (6.1)	3 (5.4)	16 (6.4)
*Klebsiella oxytoca*	13 (10.1)	8 (12.1)	6 (10.7)	27 (10.8)
*Klebsiella pneumoniae* spp.	22 (17.1)	8 (12.1)	8 (14.3)	38 (15.1)
*Kluyvera* spp.	–	1 (1.5)	–	1 (0.4)
*Raoultella ornithinolytica*	2 (1.6)	3 (4.5)	–	5 (2.0)
*Serratia ficaria*	–	–	2 (3.6)	2 (0.8)
*Serratia liquefaciens*	2 (1.6)	–	–	2 (0.8)
*Serratia marcescens*	3 (2.3)	–	1 (1.8)	4 (1.6)
*Serratia odorifera*	2 (1.6)	–	–	2 (0.8)
*Serratia plymuthica*	–	1 (1.5)	–	1 (0.4)
Environmental bacteria				
*Acinetobacter baumannii/lwoffii*	4 (3.1)	2 (3.0)	–	6 (2.4)
*Aeromonas hydrophila/caviae/salmonicida*	2 (1.6)	2 (3.0)	5 (8.9)	9 (3.6)
*Burkholderia cepacia*	7 (5.4)	5 (7.6)	3 (5.4)	15 (6.0)
*Chryseobacterium indologenes*	4 (3.1)	–	1 (1.8)	5 (2.0)
*Delftia acidovorans*	–	2 (3.0)	–	2 (0.8)
*Pantoea* spp.	3 (2.3)	1 (1.5)	1 (1.8)	5 (2.0)
*Pasteurella pneumotropica*^b^	1 (0.8)	1 (1.5)	–	2 (0.8)
*Pasteurella* spp.	2 (1.6)	–	–	2 (0.8)
*Proteus mirabilis*	2 (1.6)	–	–	2 (0.8)
*Pseudomonas aeruginosa*	9 (7.0)	2 (3.0)	1 (1.8)	12 (4.8)
*Pseudomonas fluorescens*	2 (1.6)	–	2 (3.6)	4 (1.6)
*Pseudomonas luteola*	4 (3.1)	7 (10.6)	6 (10.7)	17 (6.8)
*Pseudomonas oryzihabitans*	1 (0.8)	–	–	1 (0.4)
*Pseudomonas putida*	1 (0.8)	1 (1.5)	–	2 (0.8)
*Rhizobium radiobacter*^c^	2 (1.6)	–	–	2 (0.8)
*Sphingobacterium* spp.	1 (0.8)	1 (1.5)	–	2 (0.8)
*Sphingomonas paucimobilis*	–	1 (1.5)	–	1 (0.4)
*Stenotrophomonas maltophilia*	9 (7.0)	6 (9.1)	6 (10.7)	21 (8.4)
*Vibrio* spp.	–	1 (1.5)	1 (1.8)	2 (0.8)
Unidentified	–	–	1 (1.8)	1 (0.4)

The isolates were identified using API RapiD20E or API 20 NE.

^a^ Nowadays classified as *Klebsiella aerogenes*.

^b^ Nowadays classified as *Rodentibacter* spp.

^c^ Nowadays classified as *Agrobacterium tumefaciens*.

### Antibiotic resistance

Antibiotic resistance using the disc diffusion method was generally found for the majority of the isolates against AML, AMC, AMP, DA, DO, E, Ox, P, and TE, all commonly used in dentistry (Tables 4, 5). Also, frequent resistance was found against CFR, CL, and XM. There was a large variation in susceptibility among the different isolates with regard to CAZ, CFT, CTX, NOR, and CN. The majority of the isolates were, however, susceptible to CIP.

**Table 4:** Antibiotic resistance, screened with disc diffusion method, among *Enterobacteriaceae* (enteric bacteria) isolated from the oral cavity in patients with severe periodontitis, peri-implantitis, or oral mucosal lesions.

Antibiotics	*Citrobacter* spp. *N = *14 (%)	*Enterobacter* spp. *N = *24 (%)	*Escherichia coli* *N = *16 (%)	*Klebsiella oxytoca* *N = *27 (%)	*Klebsiella pneumoniae* *N = *38 (%)	*Raoultella ornithinolytica* *N = *5 (%)	*Serratia* spp. *N = *11 (%)
Commonly used in dentistry							
Amoxicillin (AML)	14 (100)	24 (100)	16 (100)	27 (100)	37 (100)^d^	5 (100)	11 (100)
Amoxicillin + Clavulanic acid (AMC)	13 (92.9)	22 (91.7)	15 (93.8)	23 (85.2)	31 (83.8)^d^	5 (100)	11 (100)
Ampicillin (AMP)	14 (100)	24 (100)	16 (100)	26 (100)^a^	37 (100)^d^	5 (100)	11 (100)
Clindamycin (DA)	13 (100)^d^	23 (95.8)	16 (100)	27 (100)	35 (100)^e^	5 (100)	9 (90.0)^d^
Doxycycline (DO)	14 (100)	23 (95.8)	15 (93.8)	23 (85.2)	36 (97.3)^d^	5 (100)	9 (81.8)
Erythromycin (E)	13 (100)^d^	22 (95.7)^d^	16 (100)	27 (100)	35 (100)^e^	5 (100)	11 (100)
Oxacillin (Ox)	11 (84.6)^d^	22 (95.7)^d^	16 (100)	27 (100)	34 (97.1)^e^	5 (100)	9 (90.0)^d^
PenicillinV (P)	13 (100)^d^	22 (95.7)^d^	16 (100)	27 (100)	35 (100)^e^	5 (100)	10 (100)^d^
Tetracycline (TE)	13 (100)^d^	22 (95.7)^d^	16 (100)	26 (96.3)	35 (100)^e^	5 (100)	9 (90.0)^d^
Cephalosporins							
Cefadroxil (CFR)^a^	14 (100)	24 (100)	16 (100)	27 (100)	34 (97.1)^e^	5 (100)	10 (100)^d^
Cephalexin (CL)^a^	14 (100)	24 (100)	16 (100)	27 (100)	35 (100)^e^	5 (100)	10 (100)^d^
Cefuroxime (XM)^b^	9 (69.2)^d^	18 (78.3)^d^	13 (81.3)	20 (74.1)	28 (80.0)^e^	5 (100)	9 (90.0)^d^
Ceftazidime (CAZ)^c^	3 (21.4)	6 (25.0)	1 (6.3)	4 (14.8)	1 (2.7)^d^	0	0
Ceftibuten (CFT)^c^	2 (15.4)	6 (25.0)	1 (6.3)	2 (7.4)	6 (16.2)^d^	0	1 (9.1)
Cefotaxime (CTX)^c^	2 (15.4)	6 (25.0)	3 (18.8)	2 (7.4)	1 (2.7)^d^	1 (20.0)	0
Fluoroquinolones							
Ciprofloxacin (CIP)	0	1 (4.2)	1 (6.3)	0	1 (2.7)^d^	0	1 (9.1)
Norfloxacin (NOR)	3 (21.4)	9 (37.5)	4 (25.0)	10 (37.0)	14 (37.8)^d^	1 (20.0)	4 (36.4)
Aminoglycosides							
Gentamycin (CN)	3 (21.4)	7 (29.2)	2 (12.5)	10 (37.0)	14 (37.8)^d^	2 (40.0)	2 (18.2)

Reported are the number of isolates not susceptible to the antibiotics tested.

^a^ First-generation cephalosporins.

^b^ Second-generation cephalosporins.

^c^ Third-generation cephalosporins.

^d^ Data from 1 strain is missing.

^e^ Data from 3 strains is missing.

**Table 5:** Antibiotic resistance, screened with disc diffusion method, among environmental bacteria harvested from the oral cavity in patients with severe periodontitis, peri-implantitis, or oral mucosal lesions.

Antibiotics	*Acinetobacter baumannii/lwoffii* *N = *6 (%)	*Aeromonas* spp. *N = *9 (%)	*Burkholderia cepacia* *N = *15 (%)	*Chryseobacterium indologenes* *N = *5 (%)	*Pantoea* spp. *N = *5 (%)	*Pasteurella* spp. *N = *4 (%)	*Pseudomonas* spp. *N = *36 (%)	*Stenotrophomonas maltophilia* *N = *21 (%)
Commonly used in dentistry								
Amoxicillin (AML)	6 (100)	9 (100)	15 (100)	5 (100)	5 (100)	3 (75.0)	34 (100)^e^	21 (100)
Amoxicillin + Clavulanic acid (AMC)	6 (100)	7 (77.8)	14 (93.3)	5 (100)	3 (60.0)	3 (75.0)	32 (91.4)^d^	21 (100)
Ampicillin (AMP)	6 (100)	9 (100)	15 (100)	5 (100)	5 (100)	3 (75.0)	35 (100)^d^	21 (100)
Clindamycin (DA)	6 (100)	8 (88.9)	14 (93.3)	3 (60.0)	5 (100)	4 (100)	32 (97.0)^f^	21 (100)
Doxycycline (DO)	6 (100)	9 (100)	14 (93.3)	5 (100)	5 (100)	3 (75.0)	30 (88.2)^e^	19 (90.5)
Erythromycin (E)	6 (100)	1 (11.1)	15 (100)	5 (100)	5 (100)	4 (100)	33 (100)^f^	21 (100)
Oxacillin (Ox)	6 (100)	9 (100)	15 (100)	5 (100)	5 (100)	3 (75.0)	32 (97.0)^f^	21 (100)
PenicillinV (P)	6 (100)	9 (100)	15 (100)	5 (100)	5 (100)	3 (75.0)	33 (100)^f^	21 (100)
Tetracycline (TE)	6 (100)	9 (100)	15 (100)	5 (100)	5 (100)	4 (100)	33 (100)^f^	21 (100)
Cephalosporins								
Cefadroxil (CFR)^a^	6 (100)	9 (100)	15 (100)	5 (100)	5 (100)	4 (100)	33 (100)^f^	20 (95.2)
Cephalexin (CL)^a^	6 (100)	9 (100)	15 (100)	5 (100)	5 (100)	4 (100)	33 (100)^f^	21 (100)
Cefuroxime (XM)^b^	6 (100)	6 (66.7)	14 (93.3)	5 (100)	4 (80.0)	4 (100)	28 (84.8)^f^	17 (81.0)
Ceftazidime (CAZ)^c^	3 (60.0)^d^	1 (11.1)	3 (20.0)	2 (40.0)	1 (20.0)	2 (50.0)	6 (17.6)^e^	10 (47.6)
Ceftibuten (CFT)^c^	4 (66.7)	1 (11.1)	4 (26.7)	4 (80.0)	2 (40.0)	2 (50.0)	18 (52.9)^e^	16 (76.2)
Cefotaxime (CTX)^c^	5 (83.3)	1 (11.1)	3 (20.0)	4 (80.0)	2 (40.0)	2 (50.0)	17 (48.6)^d^	12 (57.1)
Fluoroquinolones								
Ciprofloxacin (CIP)	1 (16.7)	0	1 (6.7)	0	0	0	0^e^	1 (4.8)
Norfloxacin (NOR)	5 (83.3)	4 (44.4)	10 (66.7)	5 (100)	2 (40.0)	2 (50.0)	17 (50.0)^e^	17 (81.0)
Aminoglycosides								
Gentamycin (CN)	2 (33.3)	1 (11.1)	3 (20.0)	3 (60.0)	1 (20.0)	0	9 (26.5)^e^	7 (33.3)

Reported are the number of isolates not susceptible to the antibiotics tested.

^a^ First-generation cephalosporins.

^b^ Second-generation cephalosporins.

^c^ Third-generation cephalosporins.

^d^ Data from 1 strain is missing.

^e^ Data from 2 strains is missing.

^f^ Data from 3 strains is missing.

In total 79 strains showed resistance to either CTX and/or CAZ. Of these 79 isolates, 74 were tested with E-test ESBL (5 were lost during storage), and 10 (13.5% of the tested isolates) were confirmed ESBL positive (Table 6). Four of these isolates were *Stenotrophomonas* species. One of the *Enterobacteriaceae* isolates, an *E. coli* isolate, was confirmed ESBL positive with the E-test ESBL. Five isolates were non-determinable using the E-test ESBL and also cefepime/cefepime + clavulanic acid, of which two were *Cryseobacterium indologenes*. Of the ten ESBL positive strains, five were isolated from mucosal lesion, four from periodontitis, and one from peri-implantitis.

**Table 6:** ESBL-positive strains isolated from the oral cavity in patients with severe periodontitis, peri-implantitis, or oral mucosal lesions, using the E-test ESBL for confirmation.

Resistant strains	Number of strains tested with E-test ESBL	ESBL−	Non-determinable	ESBL+
*Enterobacteriaceae* (enteric bacteria)				
*Citrobacter* spp. (%)	3	3 (100)	0	0
*Enterobacter* spp. (%)	7	7 (100)	0	0
*Escherichia coli* (%)	2	1 (50)	0	1 (50)
*Klebsiella* spp. (%)	7	6 (86)	1 (14)	0
*Raultella ornithinolytica* (%)	1	1 (100)	0	0
Environmental bacteria				
*Acinetobacter* spp. (%)	5	4 (80)	0	1 (20)
*Burkholderia cepacia* (%)	5	5 (100)	0	0
*Cryseobacterium indologenes* (%)	4	1 (25)	2 (50)	1 (25)
*Pseudomonas* spp. (%)	17	15 (88)	1 (6)	1 (6)
*Stenotrophomonas maltophilia* (%)	14	10 (71)	0	4 (29)
Other^d^ (%)	9	6 (67)^a^	1 (11)^b^	2 (22)^c^
Total number of strains (%)	74^d^	59 (80)	5 (7)	10 (14)

^a^ *Aeromonas* spp., *Pasteurella pneumotropica* (2 isolates), *Rhizobium radiobacter*, *Sphingobacterium spiritivorum*, *Sphingomoonas paucimobilis*.

^b^ *Pantoea* spp.

^c^ *Pantoea* spp., *Sphingobacterium* spp.

^d^ Strains that showed resistance to either CTX and/or CAZ were tested.

## Discussion

In this study 251 strains of aerobic Gram-negative bacilli (AGNBs) that showed at least moderate growth and were associated with oral inflammatory sites (mucosal lesion, periodontitis, or peri-implantitis) were isolated and identified. The isolates represented a great variation on genus/species level and belonged to various groups (Families): including *Enterobacteriaceae, Pseudomonadaceae* and other environmental bacterial families*.* Even if some species occurred more frequently than others, the important observation here was that the isolated AGNBs represented a broad spectrum of Gram-negative aerobic bacilli normally found in the gastrointestinal tract or in the human environment. They are not normally colonizing the human oral cavity and are considered non-oral (30). They are all easy-growing bacteria on ordinary nutrient agar. They were selected and identified when present in the predominant microbiota in order to avoid transient and occasional occurrence of AGNBs. In 32 samples two or three AGNB strains were present concomitantly, indicating that the establishment of these bacteria in the oral cavity involves a high degree of randomness although a number of determinants may increase the risk for their establishment (See below). In the peri-implantitis group, the majority of the samples were detected during the follow-up period after treatment, indicating a resistance against periodontal treatment in general as previously noted by van Winkelhoff et al. (13).

### Patients and samples

The isolates used in this study were collected from incoming samples of dentists in the western region of Sweden and we were not able to identify patients (if any) with immune-compromised condition among patients with periodontitis. In the 100 peri-implantitis patients treated and followed no immune-compromised patients were included (23). Only 12 samples taken from mucosa were from immune-compromised individuals, which confirms that AGNBs sometimes occur in local opportunistic conditions in the oral cavity as reported earlier (6, 12) as well as in the medical area in general (1, 2, 4). It must be emphasized that the vast majority of AGNBs included in this study come from local inflammatory lesions in otherwise healthy patients.

### Bacterial identification

A detailed identification of the AGNBs was performed in this study since very few such studies have previously been carried out. Further, substantial taxonomic changes have been performed through the years and an updated description of the oral AGNBs species found in this study is warranted.

*Enterobacteriaceae* or enteric rods are characterized by their ability to ferment carbohydrates e.g., glucose (Vogues Proskauer test) and lactose. Their main habitat is the human gastrointestinal tract. They are also commonly present in various environments, such as food and water, and referred to as fecal contaminations. Enteric genera/species (*Enterobacter, E. coli, Klebsiella, Citrobacter, Serratia* species) are considered low virulent but are frequently occurring in immunocompromised and multi-diseased patients as opportunistic infections. They are considered non-oral and should be of concern when they establish as predominant in the oral cavity. It is also noticed that enteric rods frequently occur in the transient microbiota due to poor hygiene, fecal-oral route contacts, nail-biting, animal contacts, and food and water contamination. The most frequently identified enteric rods in this and other studies (31) are *Klebsiella* spp. (*K. pneumoniae* and *K. oxytoca*). Both species are important medical pathogens, especially in respiratory tract infections, pneumonia, and sepsis (2–4). The main virulence factor of *Klebsiella* species is the production of a capsule, which makes them resistant against phagocytosis and intracellular killing by leukocytes. Sepsis due to *Klebsiella* spp. has often a fatal outcome (32). Their impact in oral dysbiosis cases, however, is unclear and only a few reports have identified *Klebsiella* isolates to species level (6, 13, 14). This study confirms that *Klebsiella* is one of the most frequent AGNBs that can establish, given the opportunity, in the human oral cavity, not necessarily in systemically immune-compromised individuals but also in cases of a locally compromised mucosal and/or periodontitis/peri-implantitis lesions, in otherwise healthy patients.

While *Klebsiella* species account for the major number of enteric rods in oral dysbiotic lesions, other enterics such as *Citrobacter* spp. *Enterobacter* spp., *E. coli*, and *Serratia* spp. were also frequently occurring, as has been previously reported (8, 12, 13, 33). The most frequent genera, *Enterobacter* and *Escherichia* are easily identified on MacConkey agar by its ability to ferment lactose and are in some studies referred to as coliforms or just enterics or enteric rods (10, 34). *Enterobacter aerogenes* has changed its taxonomic position and is nowadays referred to as *Klebsiella aerogenes* (35). This species has previously been found in two chronic periodontitis cases (34). In our investigation, *Citrobacter* spp*.* accounted for 14 isolates growing in moderately rich numbers, (*Citrobacter freundii* 10 isolates). These have previously only sporadically been reported from the oral cavity (13). *Citrobacter* spp. forms typical yellow colonies on nutrient agar. They are reported as significant medical pathogens in neonates and immune-compromised patients (36).

Similarly, *Serratia* spp. have sporadically been reported from the human oral cavity (13, 14, 34). They are also considered medical pathogens and have long been known for causing nosocomial infections in neonates and in immune-compromised patients (37).

Some enterics were more occasional in the present study such as *Cedecea, Cronobacter, Kluyvera, Proteus* and *Pantoea*. Some *Enterobacteriaceae, Cedecea davisae* 1 isolate, *Cronobacter* spp. 1 isolate, *Proteus mirabilis* 2 isolates, *Kluyvera* spp. 1 isolate and *Pantoea* spp. 5 isolates were found only in a few cases and are apparently rarely occurring in the oral cavity. *Pantoea agglomerans, Klyuvera* spp. and *P. mirabilis* have been reported occasionally (13, 34), while *Cronobacter* spp. and *C. davisae* have not previously been reported in human oral microbial samples.

Non-fermenting oxidase and catalase-positive AGNBs detected in opportunistic infections are commonly identified as the family *Pseudomonadaceae*. They are generally strictly aerobic and do not grow anaerobically (although they may survive). They are present in various environments, including the gastrointestinal tract but are not considered primarily as enterics but environmental bacteria with an important ability to form biofilms (38). Studies using molecular identification report *Pseudomonas* in low numbers in dental plaque material, but although they are easily growing bacteria they are normally not detected in oral samples using culture. They are multidrug resistant and of particular concern in the respiratory tract and lung infections in compromised patients (Cystic fibrosis, COPD, intubation), or generally in hospitalized patients. *Pseudomonas* species (most frequently *P. aeruginosa* and *P. luteola*) constitutes one of the major groups of AGNBs isolated from oral inflammatory dysbiotic conditions in this study, which is in agreement with a previous report (8).

*S. maltophilia*, a closely related genus to *Pseudomonas* and previously termed as *Pseudomonas maltophilia* is now classified as its own family, *Stenotrophomanadacea*. It is described as an emerging global pathogen (39). It has however previously rarely been reported from the oral cavity, probably due to its inclusion into the genus *Pseudomonas*. *S. maltophilia* in oral mucosal infections have however been presented earlier in case reports (40).

Previously *Burkholderia* or *Burkholderia* complex was similarly classified into the *Pseudomonas* family but constitutes now its own family. Winkelhoff et al. reported *Burkholderia* in a few periodontitis cases (13).

*Acinetobacter* spp. (family *Acinetobacter*) constitutes the third major group of AGNBs isolated from dysbiotic sites in the oral cavity, besides *Enterobacteriaceae* and *Pseudomonadaceae* in this and other publications (13, 41, 42). They are mainly reported as *Acinetobacter baumannii* and are colonizing periodontal sites (43), while reports of its presence from oral mucosal lesions are lacking. We found 4 out of 6 *Actinobacter* spp. to be isolated from oral mucosal lesions. These have not been reported in oral samples from healthy individuals, including microbiome studies with non-cultural methods, but are associated with recurrent aphthous stomatitis (44). *Acinetobacter* are strictly aerobic and non-fermentative Gram-negative rods, which are widely distributed in nature and in food and water. Furthermore, they are multidrug resistant hospital pathogens (45).

*Aeromonas* (family *Aeromonaceae*) are strictly aerobic and non-fermentative Gram-negative rods that are present in soil and water. They are reported as emerging pathogens with increasing significance in public health (46). The reports of *Aeromonas* species establishment in the oral cavity are rare although van Winkelhoff et al. reported 4 cases from periodontitis (both before and after periodontal treatment) (13). In the present study we can add another 9 cases isolated from mucosal lesions, periodontitis and peri-implantitis. In view of the increasing problems for public health, *Aeromonas* should be identified when they occur in oral dysbiosis cases.

*Pasteurella* species (e.g., *P. pneumotropica*) are considered zoonotic pathogens, which occasionally occur in infection in humans (47). Its presence in the human oral cavity has previously been reported in one case (13).

A number of environmental Gram-negative aerobic species were detected only in a few cases in this investigation. *R. radiobacter, Sphingomonas paucimobilis, Sphingobacterium* spp., and *Delphia acidovorans*, have been reported to be involved in nosocomial infections but not previously reported from the oral cavity. Other species, to our knowledge not previously found in high numbers from oral lesions, include *Chryseobacterium indologenes* belonging to the family *Weeksellaceae*. It is a low virulent AGNB, which is present in soil and water and occasionally in the human intestine, but rarely in infections, although there is a report on *C. indologenes* in nursing home-associated infections (48).

*Sphingobacterium* spp. that are present in nature were identified in two cases in this study. *Sphingobacterium* spp. are rarely causing infections in humans although this bacterial genus has been reported as a respiratory tract infection in patients with cystic fibrosis (49), however with an unclear etiological role. Further, we found one isolate of *Sphingomonas paucimobilis*, a Gram-negative non-fermenting bacillus, being a wide-spread cause of nosocomial infections (50). *S. paucimobilis* is an opportunistic pathogen, that takes advantages of underlying conditions and disease. This is the first report of *S. paucimobilis* found in the human oral microbiota.

*R. radiobacter* was isolated in two cases. *Rhizobium* belongs to the family *Rhizobiaceae* and may cause diseases in plants. It is occasionally reported in humans. Winkelhoff et al. reported 6 cases of *R. radiobacter* in periodontitis patients after periodontal debridement but none before treatment (13). Two isolates of *Vibrio* spp. were also found in the present study in patients with oral inflammatory dysbiotic conditions. Vibrios are well-known motile Gram-negative bacillus that commonly cause skin -and ear infections (51). They are aquatic bacteria that prefer seawater with moderate salinity. We were not able to specify the two isolates to species level and report them here only as *Vibrios* spp.

### Antibiotic resistance

AGNBs are generally multidrug resistant, which is an emerging problem generally in medicine worldwide. This study also confirms that this is the case also for isolates found predominantly in inflammatory sites of the oral mucosa, periodontitis, and peri-implantitis. A broad diversity of isolated AGNBs, including a number of genera of enterics and environmental bacteria, indicates that this is due to a general intrinsic resistance and tolerance against various antimicrobial substances present in nature. AGNBs also frequently uptake resistant genes by horizontal transfer of plasmids and contribute seriously to the spread of resistance to other AGNB genera and species. A third explanation for the resistance against penicillins is by the presence of the outer membrane in all Gram-negative bacteria, which protects the synthesis of the target proteoglycan of the bacterial cell wall during growth. Finally, AGBŃs are excellent producers of various beta-lactamases, which makes them resistant against penicillins and cephalosporins. Of particular importance is the low *in vitro* susceptibility for penicillins, including Ox, AMP, AML, and AMC, since they are used massively and blindly by dentists in combating oral infections, periodontitis, and peri-implantitis. *K. pneumoniae* (83.8% resistance) and *K. oxytoca* (85.2% resistance) showed some susceptibility for AMC in our study, while Jepsen et al. (31) found a 100% resistance of oral *K. pneumoniae* and *K. oxytoca* against AML but a higher susceptibility for AMC and DO. They also found a high resistance among *Enterobacter aerogenes* (now *Klebsiella aerogenes*) and *Serratia* species as was found in the present study. Especially alarming for dentistry is the overall resistance of almost all isolates against AML and AMC, two antibiotics that are, except for a few countries, common as adjunct in the treatment of severe periodontitis and peri-implantitis (27).

In a systematic review by Teughels (52), a modest mean benefit of approximately 0.4 mm in Probing Pocket Depth reduction with a follow-up period up to 1 year was reported for adjunctive use of antibiotics in the treatment of periodontitis. According to the European Federation of Periodontology clinical practice guidelines (53) adjunctive systemic antibiotics should not be used routinely but may be considered for specific patient categories (e.g., generalized periodontitis stage III in young individuals). In the treatment of peri-implantitis, adjunctive use of antibiotics is even more common but the evidence for such use is sparse (54). Overall, the initial additive effect of antibiotics in treatment of periodontal and peri-implant diseases is believed to be fading over time and this has also been reported in some studies (54, 55). The potential benefits of using antibiotics in the treatment of oral conditions should be balanced against the side effects of their use. AGNB in oral lesions should be recognized as potential reservoirs for development and spread of antibiotic resistance. Cephalosporins are less used in dentistry than penicillins (27), although they are alternatives for upper respiratory infections with AGNBs showing a general susceptibility for CAZ, CFT, or CTX. However, since resistance against the third generation of cephalosporins constitutes the phenotypic identification of ESBL, diagnostics is necessary. ESBL was primarily detected among *E. coli* and *Klebsiella* species, but was later found among other enterics as well as other AGNBs (17). In the present study, one enterics was confirmed to be ESBL among the 10 strains that were confirmed ESBL positive (Table 6). ESBL was primarily (4 strains) found among *S. maltophilia*. Five strains were non-determinable using E-test ESBL, which indicates that a final ESBL identification should be based on the presence of resistant genes.

The *Pseudomonas* isolates showed a multidrug resistance including AML, AMC, and first and second generation cephalosporins. Notably only 1 of the tested 17 strains were phenotypically defined as ESBL isolates. ESBL isolates among *Pseudomonas* have previously been described in the literature (16). ESBL and carbapenem resistance is reported as a growing problem in respiratory infections of *Acinetobacter* in hospitals (56). Further, some isolates (*C. indologenes*, *Sphingobacterium*) were also registered as ESBL positive, a finding that is new.

The presence of one ESBL isolate in the human oral cavity has previously been reported in Norway (19). One other study, however, did not detect any ESBL-encoding genes (15). A low prevalence of multidrug-resistant bacteria, including ESBL (using ESBL ChromeAgar), was also observed among undergraduate dental students in dental schools in Italy, The Netherlands, and Sweden (20). The use of ESBLChromeAgar is developed for detection of ESBL strains among enterics (*E. coli* and *Klebsiella*), while the relevance to use ESBLChromeAgar for other genera such as *Pseudomonas* and *Stenotrophomonas* is more uncertain. The finding of ESBL strains isolated from the oral cavity should be of concern for the dental clinic. The hygiene measures should always be optimal so that the spread of these bacteria is minimized. It is also a concern for the general medical care and hospital that oral cavity in patients, especially those that have oral infectious sites, also constitute reservoirs for multidrug resistance including ESBL for translocation into other body sites.

Although CIP, NOR, and other fluoroquinolones (e.g., Moxifloxacin) (57) seems to be a drug of choice for infections by AGNBs, its use for dental infections is controversial. CIP was suggested as the drug of choice in the treatment of periodontitis when AGNBs were present already 1990 by Slots et al. (58) and was recently further launched in combination with metronidazole in recalcitrant periodontitis cases (59). It should be emphasized that CIP should only be used after microbiological diagnosis and identification of the specific target, such as AGNB. It should also be noted that there is a recommended restriction on the use of fluoroquinolones by the European Medicines Agency (PRAC, European Medicines Agency, 2018), that express a general concern of using fluoroquinolones uncritically and against benign infections due to rare but potentially long-lasting side effects.

### Clinical significance of oral AGNBs

The role and significance of AGNB establishment in the human oral cavity is unclear and controversial. The occurrence in oral mucosal lesions has been reported in numerous studies (8, 10, 12), however their significance for the lesion is undecided. Lesions like stomatitis, glossitis and other local mucositis with overgrowth of AGNBs or other opportunistic microorganisms (*Candida* species, enterococci and *S. aureus*) (12) are often associated with pain, burning sensations, complaints and discomfort, which indicates that they are of importance although they are not invasive and termed “infection”. Dysbiosis is thus a more appropriate term.

The significance of AGNBs in periodontitis and peri-implantitis is also controversial (13, 14, 24, 34, 60). In cited publications as well as in the present study, the samples were taken from deep periodontal pockets with paper points and thus were claimed to represent a “sub-gingival sample” from a niche that is characterized for its low redox potential and a microbiota predominated by strictly anaerobic species (*P. gingivalis*, *Prevotella* spp., *Fusobacterium* spp. and spirochetes) (61). Since the majority of the AGNB isolates in this and other studies are facultative anaerobic or even strictly aerobic bacteria, it is argued that these bacteria do not successfully compete and establish in deeper periodontal/peri-implantitis pockets. Thus, ANGBs are unlikely to contribute significantly to disease progression. It is more probable that they colonize the oral mucosal lining, including the gingiva, if the conditions are favorable by locally compromised conditions such as bad oral hygiene and inflammation, creating an imbalance within the oral microbiota and between the mucosal microbiota and the host response, resulting in an overgrowth, a dysbiosis. This dysbiosis may have consequences both locally, by severely aggravating the inflammation and exacerbating with symptoms, ulcerations, and risk for invasion and infection. So, dysbiosis, whether it occurs in mucosal lesions, periodontitis or peri-implantitis, should primarily be viewed as overgrowth of AGNBs colonizing the surface. Dysbiosis is usually associated with inflammation, and should be controlled with oral hygiene measures, not be treated with antibiotics.

The frequent and predominant occurrence of multidrug resistant AGNBs is a major concern in the treatment of oral dysbiotic conditions/infections. Antibiotics are recommended to be used as an adjunct to non-surgical debridement or surgeries, and oral hygiene measures in the treatment of periodontitis and peri-implantitis in many countries (52). Due to the unclear etiological role of specific “periodontal pathogens” for progression and thereby undefined targets for the antibiotic activity, extended broad-spectrum antibiotic combinations, e.g., amoxicillin (with or without clavulanic acid) together with metronidazole are used (52). Few studies have considered the risk of treatment failures and superinfections in the case of multidrug resistant AGNBs (62).

What makes the AGNB to colonize in the human oral cavity and be present in inflammatory dysbiotic cases as in the present study is not known. It is likely that antimicrobial treatment (using antibiotics and antiseptics) disrupts the balance by the reduction of antimicrobial sensitive microorganisms and facilitate the presence and growth of non-oral AGNBs. Winkelhoff et al. (13) reported that AGNBs were more frequent after periodontal debridement. Scannapieco et al. (63) reported no effect on AGNBs using chlorhexidine rinsing, but a reduction in Gram-positive bacteria such as *Streptococcus*, *Staphylococcus* and *Enterococcus* species. By the increasing use of antibiotics (including broad-spectrum penicillins and clavulanic acid), it is likely that the antibiotics in various forms in periodontitis and peri-implantitis therapies potentially contribute to periodontal treatment failures and superinfections which results in permanent establishment of AGNBs in the oral microbiota; and thus becomes a reservoir for spread to the respiratory tract and lungs or elsewhere in the body. This risk has been particularly emphasized in hospitalized and ventilated patients and patients with various forms of lung diseases (COPD, cystic fibrosis, and pneumonia) (64). The treatment of these inflammatory dysbiotic conditions with AGNBs should be symptomatic by oral hygiene improvement, professional tooth cleaning procedures, and local antiseptics. Due to the multidrug resistance of AGNBs, local as well as systemic antibiotics should for several reasons be avoided in these inflammatory dysbiotic conditions with questionable infectious nature. Thus, the importance of mechanical debridement, potentially in combination with surgical interventions and drainage of dysbiotic or infectious conditions, such as mucosal lesions, periodontitis and peri-implantitis, is emphasized. Additionally, the potential use of antibiotics in rare cases should be based on microbiological sampling and analysis of the lesion, for identification of the microbiological target.

This study is one of very few with an open approach where the presence of almost all cultivable bacteria is investigated and the AGNB predominant species, those with at least moderate growth of AGNB, are further analyzed with regard to antibiotic resistance. Despite the retrospective design and the lack of clinical details of patients, this investigation demonstrates that also typical non-oral species may be predominant in various oral lesions. Their presence in high numbers may possibly influence the pathogenesis and/or the lack of resolution of the various oral conditions.

### Conclusions

AGNBs may occur as predominant in oral inflammatory lesions such as mucositis, periodontitis, and peri-implantitis. AGNBs includes enterics as well as environmental bacteria and show a broad diversity on species level although some species occur more frequently than others. They are generally multidrug resistant, and ESBL variants may occur. No clear pattern of AGNB species in the three studied inflammatory dysbiotic conditions could be identified. It is recommended not to use antibiotics without a microbiological sample and diagnosis in the treatment of oral mucosal lesions, periodontitis, and peri-implantitis due to the risk of undesirable effects of AGNBs presence.

## Data Availability

The original contributions presented in the study are included in the article. Further inquiries can be directed to the corresponding author.
